# Differences in cognition, short-chain fatty acids and related metabolites in pregnant versus non-pregnant women: a cross-sectional study

**DOI:** 10.1186/s12884-022-04853-2

**Published:** 2022-07-01

**Authors:** Huijuan Luo, Wengxiang Li, Lulu Wu, Shuming Zhong, Chengrong Du, Yimeng Liu, Yating Xu, Xinyu Huang, Awol Hanan Bahru, Xiaomei Tang, Juan Zhou, Dongju Wang, Xiangying Lou, Xuefan Bin, Xiaomin Xiao

**Affiliations:** 1 Department of Obstetrics and Gynecology, The First Affiliated Hospital of Jinan University, No.601, West Huangpu Avenue, Guangzhou, Guangdong 510630 China; 2 Department of Psychology, The First Affiliated Hospital of Jinan University, Guangzhou, Guangdong 510630 China; 3 Department of Clinical Medicine, International College, Jinan University, Guangzhou, Guangdong 510630 China; 4 Shanghai Medical College, Fudan University (SMCFU), 138 Yi xue yuan Road, Shanghai, 200032 China

**Keywords:** Cognitive, Short-chain fatty acids, Pregnancy

## Abstract

**Background:**

Pregnancy induces cognitive reorganization which can lead to mental disorders. The aim of this study is to determine differences in cognitive scores, short-chain fatty acids (SCFAs) and related metabolites between pregnant and non-pregnant participants.

**Methods:**

This cross-sectional study included 67 full-term pregnant women and 31 non-pregnant women. We compared scores of mental state and cognitive assessment tests, as well as serum concentrations of SCFAs, hormones, inflammatory factors, and neurotransmitters between these groups.

**Results:**

Scores for information processing speed, immediate visual memory, motor response speed and accuracy, execution ability and verbal use ability in the pregnant group were lower than those in the non-pregnant group (*p* < 0.05 for all tests). Total serum SCFAs in the pregnant group were significantly lower than those in the non-pregnant group (*P* = 0.031). Among them, acetate and propionate were significantly decreased (*P* = 0.013 and 0.037, respectively) whereas butyrate was significantly increased (*P* = 0.035). Serum peptide YY, glucagon-like peptide-1, γ-aminobutyric acid, and dopamine showed no differences between the two groups. However, cortisol, adrenocorticotropic hormone, and acetylcholine were significantly increased in the pregnant group as compared with the non-pregnant group (*P* = 0.039, 0.016, and 0.012, respectively). Tumor necrosis factor-α was increased and interleukin-10 significantly decreased in the pregnant group (*P* = 0.045 and 0.019, respectively).

**Conclusion:**

According to our study findings, cognitive reorganization in the third trimester of pregnancy showed that both the passive storage capacity of working memory and the executive function of online information processing were decreased to varying degrees. At the same time, the changes in total SCFAs, the proportions of SCFAs and related metabolites were also detected. These changes in the internal environment may be increasing the risk of perinatal mental illness.

## Background

Pregnancy is an important period during a woman’s life that can bring about dynamic changes in cognition. Previous studies have shown that pregnancy can induce cognitive reorganization [1, 2]. Most cognitive changes in pregnant women are physiological. However, even once these end, cognitive changes in pregnancy can lead to mental disorders and other pregnancy complications, such as bipolar disorder, preeclampsia, abortion and premature delivery [3, 4]. Changes in cognition during pregnancy may persist after childbirth, affecting the physical and mental development of the mother and offspring [5, 6]. Previous studies of brain function show that short-chain fatty acids (SCFAs) may play an important role as metabolites of intestinal flora. SCFAs can change the function of neurons, glia, and the blood–brain barrier (BBB) through the pathways of metabolism, immunity, and the enteric nervous system, and supplementation with SCFAs has shown good effects in bipolar disorder and depression in animal experiments [7–10]. Pregnant women with obvious cognitive and emotional changes are often concerned about harm to the fetus caused by drugs and refuse treatment. Finding a safe and reliable method of preventing and treating such changes in pregnancy has become an urgent clinical need. By exploring the relationship and possible mechanisms of changes in SCFAs with cognition and emotions in pregnant women, we aimed to lay a foundation for finding a safe and effective method to prevent and treat pregnancy complications and perinatal psychological diseases owing to metabolic changes in SCFAs during pregnancy.

## Methods

### Aim

The aim of this cross-sectional study is to determine differences in cognitive scores, SCFAs and related metabolites between pregnant and non-pregnant participants. It provides ideas for finding an effective prevention method of psychological diseases in pregnancy in the future.

### Design and setting of the study

This project is a cross-sectional study. The study group was full- term healthy pregnant women and the control group was healthy non-pregnant women.

### Participants

In this study, the pregnant group consisted of healthy pregnant women who met the inclusion criteria and had regular prenatal examinations and normal delivery between January 2020 and June 2021 in the Obstetrics Department of the First Affiliated Hospital of Jinan University. Healthy non-pregnant women who came to our hospital for pre-pregnancy checkups or consultation were included in the non-pregnant group. All participants were enrolled in this study after signing an informed consent form.

Inclusion criteria for the non-pregnant group were as follows: Chinese nationality; aged 20–34 years; bachelor’s degree or above; long-term residence in Guangdong Province, southern China; no history of digestive, respiratory, cardiovascular, other autoimmune, endocrine, or metabolic diseases; no history of vaginitis, use of antibiotics or contraceptives within the past month; and no history of pregnancy.

Inclusion criteria for the pregnant group were as follows: single primipara; Chinese nationality; age 20–34 years; bachelor’s degree and above; long-term residence in Guangdong Province, southern China; no history of digestive, respiratory, cardiovascular, other autoimmune, endocrine, or metabolic diseases; no history of vaginitis, use of antibiotics or other drugs within the past month; and 37–40 weeks’ gestation with no pregnancy complications.

### Sample size calculation

Estimated from the pre-experiments, the total content of serum SCFAs in pregnant group is 2 μg/g and 3 μg/g in non-pregnant group, between which the standard deviation is 1 μg/g. The sample size was calculated by the method used in the literature (a = 0.05, power = 0.8) [11], and the minimum sample size was about 16 in each group.

### Demographic information

Basic data of two groups were collected, including age, body mass index (BMI) education (bachelor = 0, Master = 1, doctor = 2, postdoctoral = 3), income (<100 thousands yuan/year = 0, ≥100–200 thousands yuan/year = 1, ≥200–300 thousands yuan/year = 2, ≥300–400 thousands yuan = 3), appetite (very poor/excellent = 0–10); diet structure (It was rated on a ten-point scale from totally vegetable to totally meat).

### Cognitive and psychological assessment

The Symbol Digit Modalities Test (SDMT), Digit Span, Trail Making Test (TMT), Visual Reproduction Test (VRT), Wisconsin Card Sorting Test (WCST), and Verbal Fluency Test (VFT) were used to evaluate cognitive functions in terms of information processing speed, attention, visual memory, executive function, language, and other neurocognitive functions. These tests have been proven to be effective in the assessment of individuals with mild cognitive impairment and healthy individuals. All participants were evaluated by five medical students who had received standardized training. Assessment was conducted in a quiet environment for approximately 30 minutes, and all tests were administered to all women in the same order: first the SDMT, and then the Digit Span, TMT-A, TMT-B, VRT, WCST, and VFT. One psychologist graded the scores of cognitive assessments, and two graduate students entered all the data. All blood samples were sent to Testing Center in the same batch for testing. All measures were taken to ensure that the data were scientific and valid.

The SDMT is a symbol digit modalities test in the Chinese Revision of the Wechsler Adult Intelligence (WAIS-RC), which reflects information processing speed of individuals. The evaluation parameter is the total number of correct symbols selected within 90 seconds (0–90 points). A higher score indicates better processing speed.

The Digit Span is a part of the WAIS-RC test. The participants’ attention function and working memory span are reflected in its two parts, involving series of digits of varying length. The lowest score is 0 and the highest score in parts A and B is 14 and 28, respectively. Higher scores on all items indicate better attention and working memory.

The TMT includes part A and B, which measure the speed and accuracy of motor responses. In part A of this test, the participant is asked to draw a line connecting 25 numbers in ascending order. Part B requires drawing a line to connect alternating numbers and letters in an ascending pattern. The time needed to complete the task is measured. If an error is made, it is pointed out immediately and the participant is allowed to correct the mistake. The correction of errors and the frequency of pen lifting are included in the total completion time for the task.

The VRT is used to assess immediate visual memory. In this study, we used the Visual Reproduction test of the Wechsler Memory Scale (WMS-R) revised in China. The lowest score is 0, and the highest score is 14, with higher scores indicating better visual memory.

The WCST is a modified version of the Wisconsin Card Classification Test, comprising 4 stimulus cards and 48 response cards, used to assess executive function. A greater number of total classifications completed as well as fewer total, persistent. and non-persistent errors together indicate better executive function.

The VFT is an animal-naming test that measures executive function and verbal use. Individuals are asked to name as many different animals as possible within 1 minute. The evaluation parameters are the correct number of named animals (total score), number of repetitions, and number of errors. Higher total scores and fewer repetitions and errors indicate better executive function and verbal use.

Psychological states and sleep quality were evaluated using the Pittsburgh Sleep Quality Index (PSQI). Depression and anxiety were assessed using the General Anxiety Disorder (GAD)-7 and Patient Health Questionnaire (PHQ)-9.

### Comparison of serum SCFAs

After signing an informed consent form, 4 mL of venous blood was extracted from each participant and centrifuged at 3000 rpm for 10 minutes. The serum was then separated and stored at − 80 °C until examination. Metabolomics analysis was performed using gas chromatography-mass spectrometry (GC-MS). The serum concentrations of six common SCFAs (acetate, propionate, isobutyric acid, butyrate, isovaleric acid, and valerate) were determined in the two groups. We used a Thermo Fisher Scientific GCMS ISQ LT with the following GC-MS conditions: column temperature: 100 °C (5 min), 5 °C/min, 150 °C (0 min), 30 °C/min, 240 °C (30 min); flow rate: 1 mL/min; shunt ratio 75:1; carrier gas: helium; chromatic column: TG Wax 30 m × 0.25 mm × 0.25 μm; sampler: 240 °C; mass spectrometry: electron ionization source; bombardment voltage: 70 eV; single-ion scanning mode: quantitative ion 60, 73; ion source temperature: 200 °C; temperature of connecting wire: 250 °C. The sample pre-treatment method was as follows: add 2 ml water to the sample (1:3 phosphoric acid aqueous solution), eddy homogenization for 2 min, add 1 mL ether and extract for 10 min, centrifugation at 4000 rpm for 20 min (low temperature treatment, placed in ice water bath centrifugation); then add 1 ml ether to extract for 10 min with centrifugation at 4000 rpm. The two extracts were combined and volatilized to less than 1 mL for sample injection analysis. Finally, the external standard curve method was used for quantitative analysis.

### Comparison of serum hormones, inflammatory mediators, and neurotransmitters

Using enzyme-linked immunosorbent assay (ELISA), we detected serum levels in peripheral blood of peptide YY (PYY) and glucagon-like peptide-1 (GLP-1), which are target proteins of SCFAs; proinflammatory factors (interleukin [IL]-2, tumor necrosis factor [TNF]-α, IL-6) and anti-inflammatory factors (IL-10 and IL-22); neurotransmitters (γ-aminobutyric acid [GABA], dopamine [DA], acetylcholine [Ach]; and hormones (cortisol and adrenocorticotropic hormone [ACTH]). Standard samples and horseradish peroxidase-labeled antibodies were added to precoated microplates, and then incubated and washed thoroughly. The absorbance (optical density [OD] value) was measured at a wavelength of 450 nm using a microplate reader.

### Statistical analysis

The statistical software package SPSS 22.0 (IBM Corp., Armonk, NY, USA) was used for all data analyses. The data were summarized using descriptive statistics, with mean ± standard deviation. Single-factor analysis of variance and independent-sample *t*-tests were used to compare differences between the groups. A *P* value < 0.05 was considered to indicate statistically significant differences.

## Results

After excluding participants with incomplete data, 67 pregnant women and 31 non-pregnant women were included in the study. Among them, 26 pregnant women and 15 non-pregnant women provided blood samples after signing an informed consent form. There was no special difference between basic information (Table 1).

**Table 1:** Comparison of basic information

Basic information	Pregnancy group(*n* = 67)	Non-pregnant group(*n* = 31)	*t*	*P*
Age (year)	28.95 ± 3.65	29.45 ± 3.08	0.657	0.513
BMI	24.82 ± 2.75	25.79 ± 2.56	1.667	0.099
Education	0.64 ± 0.70	0.86 ± 0.89	1.233	0.221
Income	1.00 ± 1.08	0.93 ± 0.98	0.295	0.769
Appetite	7.60 ± 2.32	7.14 ± 2.03	0.913	0.364
Diet structure	5.00 ± 1.94	5.07 ± 1.93	0.158	0.875

Education (bachelor = 0, Master = 1, doctor = 2, postdoctoral = 3), income (<100 thousands yuan/year = 0, ≥100–200 thousands yuan/year = 1, ≥200–300 thousands yuan/year = 2, ≥300–400 thousands yuan = 3), appetite (very poor/excellent = 0–10); diet structure (It was rated on a ten-point scale from totally vegetable to totally meat)

### Comparison of psychological status and cognitive function

**Fig. 1:**
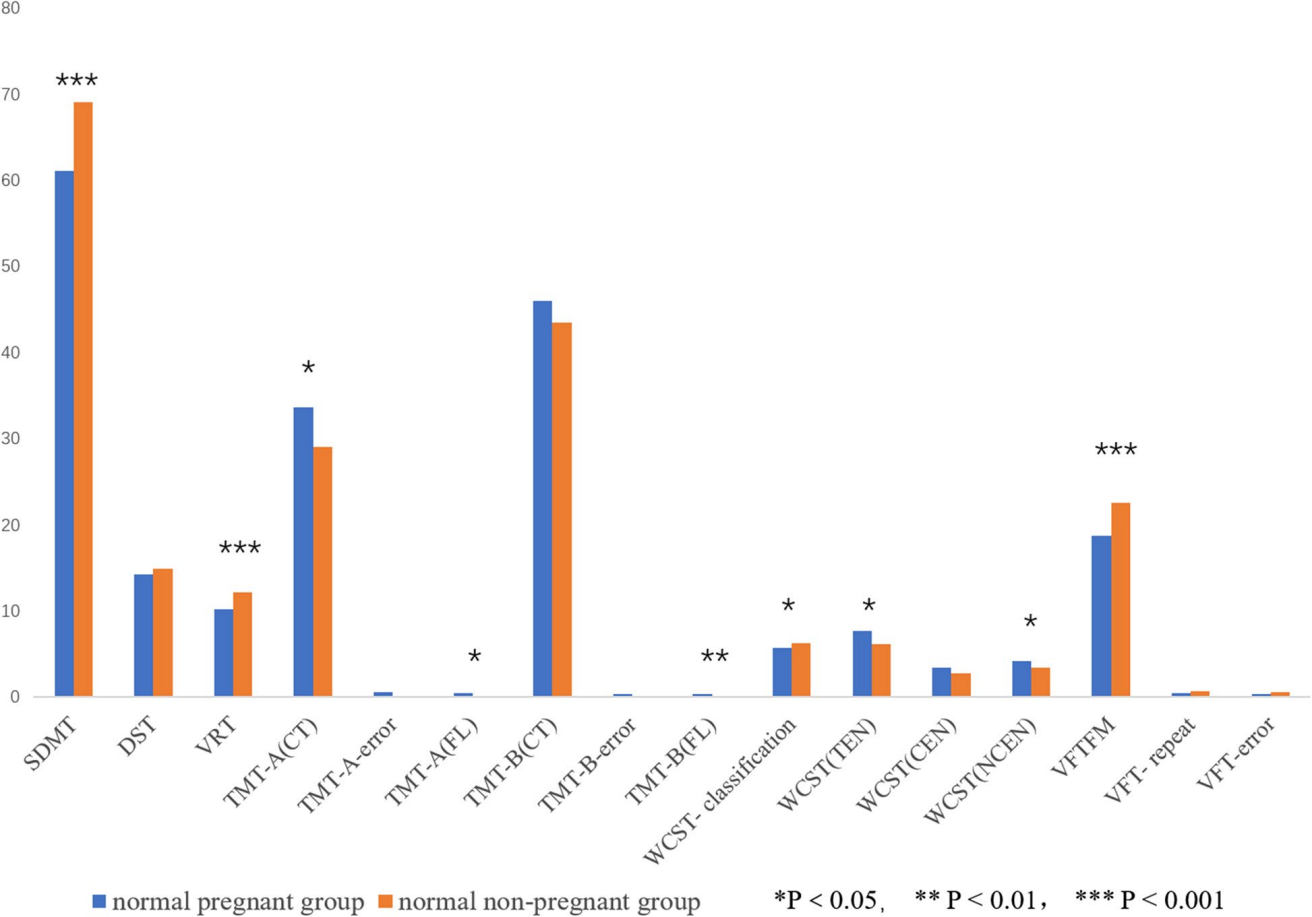
Comparison of psychological and cognitive assessment between pregnant group and non-pregnant group. SDMT: Symbol Digit Modalities Test; DST: Digit span test; TMT: Trail making test; CT: Completion time; FL: Frequency of pen lifting; VRT: Visual Reproduction Test; WCST: Wisconsin Card Sorting Test; TEN: total error number; CEN: continuous error number; NCEN: non- continuous error number; VFT: Verbal Fluency Test; VFTFM: first few minutes of the VFT. The values for TMT in non-pregnant group were not missing, but near 0. *P < 0.05, **P < 0.01, ***P < 0.001

The GAD-7, PHQ-9, and PSQI were used to assess depression, anxiety, and sleep status, respectively. No significant differences were revealed between the two groups. Comparing the cognitive function of the two groups showed that SDMT, VRT, WCST scores and the score during the first few minutes of the VFT in the pregnant group were lower than those of the non-pregnant group, and the differences were statistically significant (*t* = − 4.547, *P* < 0.001; *t* = − 5.066, *P* < 0.001; *t* = − 2.269, *P* = 0.026; *t* = − 3.953, *P* < 0.001, respectively). Scores for the TMT-A (completion time), TMT-A (frequency of pen lifting), TMT-B (frequency of pen lifting), WCST (total error number), and WCST (non-continuous error number) in the pregnant group were significantly higher than those in the non-pregnant group (*t* = 2.434, *P* = 0.017; *t* = 2.126, *P* = 0.036; *t* = 3.057, *P* = 0.003; *t* = 2.008, *P* = 0.048; *t* = 2.250, *P* = 0.027, respectively) (see Table 2, Fig. 1).

**Table 2:** Comparison of psychological cognitive assessment between pregnant and non-pregnant women

cognitive assessment	Pregnancy group (*n* = 67)	Non-pregnant group(*n* = 31)	*t*	*P*
GAD-7	3.27 ± 3.17	3.71 ± 3.06	*−0.648*	*0.519*
PHQ-9	4.02 ± 3.33	4.77 ± 3.57	*−1.026*	*0.308*
PSQI	6.13 ± 3.01	5.32 ± 2.27	*1.478*	*0.144*
SDMT	61.09 ± 7.98	69.13 ± 8.41	*−4.547*	0.000***
DST	14.28 ± 2.24	14.97 ± 2.59	*−1.343*	*0.182*
VRT	10.17 ± 2.36	12.23 ± 1.56	*−5.066*	0.000***
TMT-A (CT)	33.60 ± 8.91	29.02 ± 8.09	*2.434*	0.017*
TMT-A-error	0.625 ± 3.03	0.00 ± 0.00	*1.147*	*0.254*
TMT-A (FL)	0.46 ± 0.98	0.16 ± 0.37	*2.126*	0.036*
TMT-B (CT)	46.01 ± 10.62	43.44 ± 10.58	*1.112*	*0.269*
TMT-B-error	0.37 ± 1.30	0.03 ± 0.18	*1.995*	*0.050*
TMT-B (FL)	0.42 ± 0.71	0.10 ± 0.30	*3.057*	0.003**
WCST- classification	5.73 ± 1.24	6.23 ± 0.84	*−2.269*	0.026*
WCST (TEN)	7.67 ± 4.38	6.22 ± 2.55	*2.008*	0.048*
WCST (CEN)	3.46 ± 4.32	2.77 ± 2.96	*0.795*	*0.429*
WCST (NCEN)	4.21 ± 1.80	3.42 ± 1.06	*2.250*	0.027*
VFTFM	18.79 ± 4.25	22.58 ± 4.71	*−3.953*	0.000***
VFT- repeat	0.49 ± 0.81	0.71 ± 0.94	*−1.165*	*0.247*
VFT-error	0.35 ± 0.98	0.58 ± 1.43	*−0.911*	*0.365*

*SDMT* Symbol Digit Modalities Test, *DST* Digit span test, *TMT* Trail making test, *CT* Completion time, *FL* Frequency of pen lifting, *VRT* Visual Reproduction Test, *WCST* Wisconsin Card Sorting Test, *TEN* Total error number, *CEN* Continuous error number, *NCEN* Non- continuous error number, *VFT* Verbal Fluency Test, VFTFM: first few minutes of the VFT. *P < 0.05, **P < 0.01, ***P < 0.001

### Comparison of serum SCFAs

**Fig. 2:**
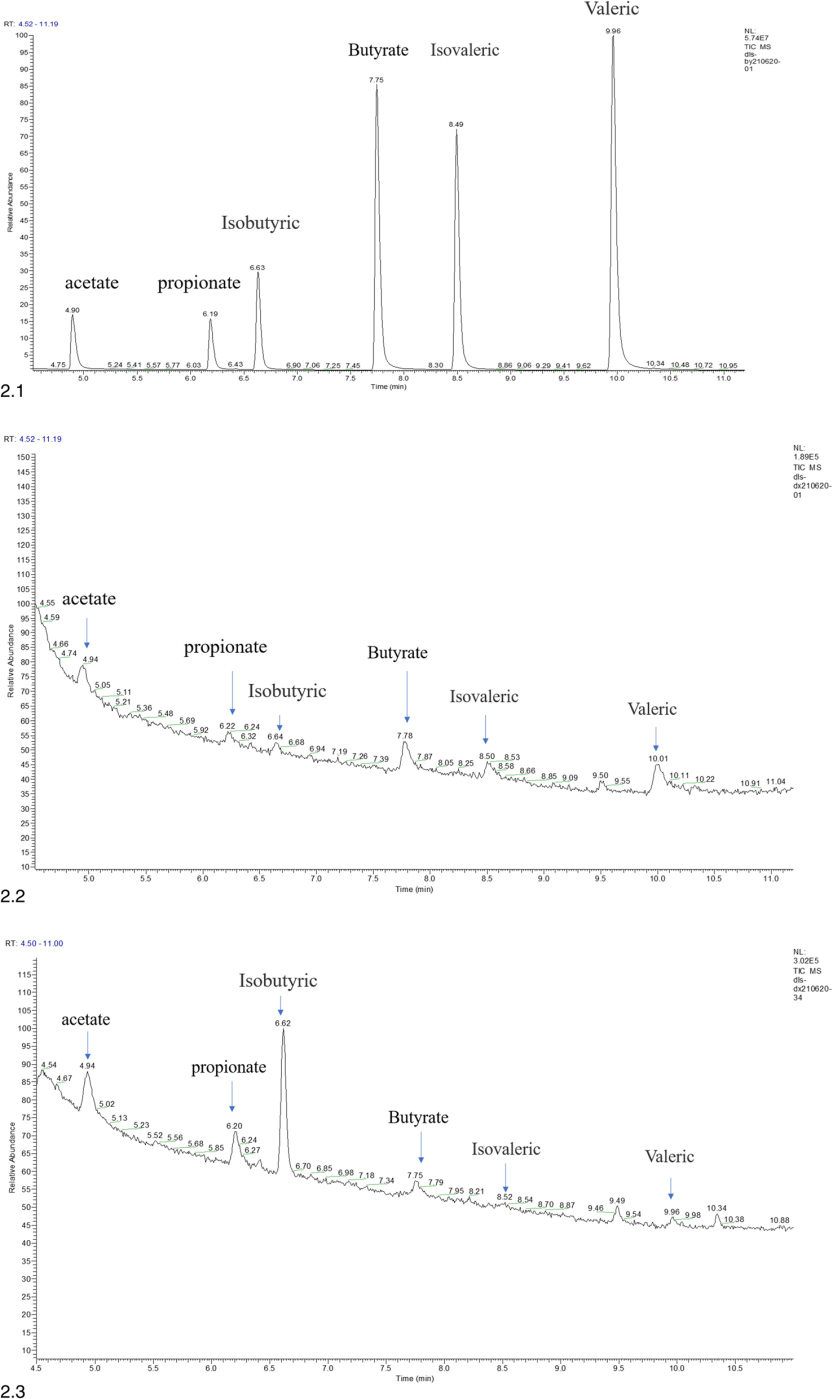
Results of gas chromatography-mass spectrometry analysis. (2.1) SCFAs test results of the standard sample; (2.2) SCFAs test results from a single representative sample of a woman in the pregnant group; (2.3) SCFAs test results from a single representative sample of a woman in the non-pregnant group

Using GC-MS, the total serum concentration of the six investigated SCFAs in the pregnant group was significantly lower than that of the non-pregnant group (*t* = − 2.244, *P* = 0.031). Among these six, serum levels of acetate, propionate, and isobutyric acid in the pregnant group showed a decreasing trend, and there was a significant difference between acetate and propionate between the two groups (*t* = − 2.609 and − 2.172; *P* = 0.013 and 0.037). In the pregnant group, butyrate, isovaleric acid, and valerate showed an increasing trend, and butyrate showed a statistically significant difference between the two groups (*t* = 2.195; *P* = 0.035). The results are shown in Table 3 and Fig. 2.

**Table 3:** Comparison of six serum SCFAs between the pregnant and non-pregnant groups

SCFA	Pregnant group (*n* = 21)	Non-pregnant group (*n* = 15)	*t*	*P*
Total SCFAs (ug/g)	1.97 ± 0.88	2.71 ± 1.10	*−2.244*	0.031*
Acetate (ug/g)	0.65 ± 0.20	0.86 ± 0.26	*−2.609*	0.013*
Propionate (ug/g)	0.55 ± 0.23	0.74 ± 0.31	*−2.172*	0.037*
Isobutyric (ug/g)	0.50 ± 0.55	0.89 ± 0.72	*−1.835*	*0.075*
Butyrate (ug/g)	0.11 ± 0.31	0.09 ± 0.33	*2.195*	0.035*
Isovaleric (ug/g)	0.81 ± 0.39	0.65 ± 0.18	*1.486*	*0.147*
Valerate (ug/g)	0.07 ± 0.32	0.06 ± 0.20	*0.593*	*0.557*

*SCFA* Short-chain fatty acid. *P < 0.05

### Comparison of serum hormones, inflammatory mediators, and neurotransmitters

Serum PYY and GLP-1 levels of SCFAs target proteins were detected by ELISA, with no significant difference between the pregnant and non-pregnant groups. There was no difference between the groups in the neurotransmitters GABA and DA, but levels of Ach were significantly higher in the pregnant group (t = 2.653, *P* = 0.012). Serum glucocorticoids cortisol and ATCH were significantly increased in the pregnant group (t = 2.152 and 2.544; *P* = 0.039 and 0.016). As for proinflammatory factors in peripheral blood, TNF-α in the pregnant group was significantly higher than in non-pregnant group (t = 2.463; *P* = 0.019), but there was no significant difference in IL-2 or IL-6 between the two groups. IL-10 was significantly reduced in the pregnant group (*t* = − 2.084; *P* = 0.045); IL-22 showed no significant difference between the groups, indicating proinflammatory changes in the blood of pregnant women. The results are shown in Table 4.

**Table 4:** Comparison of hormones, neurotransmitters, and inflammatory mediators between the two groups

SCFA-related factor	Pregnant group *N* = 21	Non-pregnant group *N* = 15	*t*	*P*
PYY (pmol/L)	12.89 ± 2.41	11.80 ± 3.48	*1.045*	*0.307*
GLP-1(pmol/L)	9.77 ± 2.56	9.19 ± 1.55	*0.786*	*0.437*
ACTH (pg/mL)	54.55 ± 12.94	44.63 ± 9.15	*2.544*	0.016*
Cortisol (nmol/L)	1168.91 ± 427.24	922.64 ± 257.02	*2.152*	0.039*
Ach (ng/ml)	28.13 ± 6.12	23.05 ± 4.97	*2.653*	0.012*
DA (pg/mL)	1026.32 ± 278.66	898.52 ± 187.21	*1.542*	*0.132*
GABA (μmol/L)	7.35 ± 2.25	6.95 ± 1.47	*0.599*	*0.553*
IL-2(pg/mL)	937.76 ± 260.53	783.12 ± 241.75	*1.808*	*0.079*
IL-6(pg/mL)	47.88 ± 16.47	49.15 ± 10.03	*−0.286*	*0.793*
TNF-a (pg/mL)	63.77 ± 17.93	50.54 ± 12.42	*2.463*	0.019*
IL-10(pg/mL)	38.34 ± 7.29	43.42 ± 7.10	*− 2.084*	0.045*
IL-22(pg/mL)	36.83 ± 12.30	31.28 ± 8.83	*1.490*	*0.145*

*PYY* Peptide YY, *GLP-1* Glucagon-Like Peptide-1, *IL* Interleukin, *TNF* Tumor necrosis factor, *GABA* γ-aminobutyric acid, *DA* Dopamine, *Ach* Acetylcholine, *ACTH* Adrenocorticotropic hormone

*P < 0.05

## Discussion

### Main finding

Cognition in pregnancy showed that both the decrease of working memory and executive function were accompanied by the decrease of total SCFAs and changes in the proportions of SCFAs which would affect the SCFAs-related factors.

Strengths of this study include that we determine differences in cognitive scores, SCFAs and related metabolites between pregnant and non-pregnant participants. It enriches the evidence for cognitive recombination during pregnancy and provides new information for the prevention of mental illness during pregnancy.

### Limitations

One limitation in our study is that this is an observational cross-sectional study design, therefore cause and effect cannot be determined. The second limitation is that no statistical tests are done to assess associations of the SCFAs and metabolites with the cognitive outcomes. The third is that generalizability of results is limited to Chinese women with a bachelor’s degree or higher and that no dietary data were collected. In future, the relationship between cognition and SCFAs during pregnancy need more in-depth study in order to find the regulatory pathway and safe method to prevent perinatal mental diseases.

### Interpretation

Studies have reported that 12% of pregnant women and 16% of parturient woman have “mental fogginess”. Multiparous women complain more about this symptom than primiparous women (31% vs. 5%) [12]. Still more women report a considerable decline in their ability to read, record details and in concentration [13, 14]. These changes are more obvious in older women and those with higher socioeconomic status [15]. Our study revealed that cognitive function changes in multiple dimensions among pregnant women, mainly on the SDMT, VRT, TMT, WCST and VET tests. This indicates that information processing speed, immediate visual memory, motor response speed and accuracy, executive ability, and verbal use ability in pregnant women may all be worse than in non-pregnant women, even with no differences in anxiety, depression, and sleep disorders between groups. Other studies have found that the decline of cognitive function is related to depression [16]. Depression affects 10–25% of pregnant women and is significantly higher than the rate in non-pregnant populations (3.2–6.9%) [3, 4]. This evidence suggests that pregnant women may be at high risk of suboptimal brain health and function, making them more vulnerable to factors that can cause perinatal mental diseases. Cognitive changes can cause pregnant women to feel they are “reacting slowly”, which may explain why pregnant women have a high risk of mental diseases.

Cognitive recombination in pregnant women is related to changes in brain structure and function during pregnancy. Shrinking of the cerebral cortex and changes on electroencephalogram and in cerebral blood flow have been found in pregnant women [17–19]. These changes are closely related to intestinal flora and metabolites, of which SCFAs—the major metabolites of intestinal flora—play an important role in the gut–brain axis [20].

Using GC-MS, we found that the total concentration of serum SCFAs in the pregnant group was significantly lower than that of the non-pregnant group and the composition was also changed: acetate and propionate were significantly decreased whereas butyrate was increased. Because the transport of butyrate in cells of the colon can be inhibited by acetate and propionate [21], the increase in butyrate during pregnancy may be the result of lower acetate and propionate levels. A reduction in total SCFAs has been detected in animal models of spontaneous depression as well as in feces of depressed patients [22, 23]. Taking three main SCFAs (acetate, propionate, and butyrate) orally can promote the maturation of microglia in germ-free mice [24]. Butyrate can inhibit lipopolysaccharide-induced body fluid regulation and depression-like behavior, such as limited eating and mobility, cognitive deficits, and social inhibition in mouse models of depression [25–28]. It is speculated that although a decrease in total SCFAs during pregnancy leads to an increased risk of perinatal mental illness, pregnant women can lower this risk by increasing their relative absorption of butyrate. Once a change in microflora or other factors occurs, the absorption of butyrate does not increase accordingly, which could induce depression.

SCFAs mainly affect brain function in the following three ways.

1. Hormone regulation. Studies have shown that SCFAs can promote the secretion of PYY and GLP-1 and inhibit the Hypothalamic-pituitary-adrenal cortex axis (HPA) response to acute psychosocial stress [29–31]. Inconsistent with the literature, we found that decreased SCFAs showed no decline in PYY and GLP-1in pregnant women as compared with non-pregnant women. Lacking the inhibition of SCFAs, increased cortisol and ACTH cause pregnant women to be more vulnerable to psychological and emotional abnormalities.
2. Immunity. SCFA-generated signals are transmitted in immune cells, maintaining anti-inflammatory and proinflammatory balance, which has an important role in the occurrence of depression [32, 33]. Microglia treated with acetate can reduce expression of IL-1 β, IL-6, and TNF-α [34, 35] whereas anti-inflammatory factors IL-10 and transforming growth factor-β can be upregulated by butyrate [36]. Thus, butyrate can be used as an anti-inflammatory agent, increase histone acetylation and interfere memory processes [37]. High levels of propionate may aggravate symptoms of autism, which can be alleviated by supplementing butyrate; this indicates that metabolite balance may be more important than levels of individual metabolites [38]. We found that TNF-α was significantly increased and IL-10 decreased in pregnant women, indicating changes that promote inflammation in full-term pregnancy. Although as an inflammatory inhibitor, butyrate was increased, it still failed to reverse the body’s proinflammatory responses. The reason may be related to the completely decreased SCFAs or inflammatory changes that exceed the adjustable range of increased physiological butyrate.
3. Neuromodulation. Through important neurotransmitters, such as GABA, DA and Ach, SCFAs can directly regulate the vagus nerve, thereby emotion and cognition [39–42]. In our study found that GABA and DA did not change during pregnancy, whereas Ach was increased. Although the mechanism remains unclear, Ach and SCFAs may coincidentally take part in the regulation of intestinal contraction and secretion [43, 44]. Abnormal gastrointestinal function is often accompanied by cognitive changes, e.g., inflammatory bowel disease, depression, and autism spectrum disorder [45].

## Conclusions

Pregnancy can lead to cognitive recombination which is manifested by reduced visual–spatial working memory and executive function. The important role of SCFAs in the gut–brain axis makes SCFAs a potential target in preventing a variety of pregnancy complications, including metabolic and psychological disorders. The decrease in total SCFAs during late pregnancy and the changes in SCFAs components, are mainly manifested as decreased acetic and propionic acid and increased butyric acid, along with the detected imbalance of inflammatory factors as well as increased Ach in peripheral blood. All those changes may increase the risk of perinatal mental diseases. The relationship between SCFAs and cognition will be determined in future studies.

## Data Availability

The datasets generated and analyzed during the present study are not publicly available due to ethical considerations but could be available from the corresponding author on request.

## Abbreviations

SCFA: Short-chain fatty acid
SDMT: Symbol Digit Modalities Test
DST: Digit span test
TMT: Trail making test
CT: Completion time
FL: Frequency of pen lifting
VRT: Visual Reproduction Test
WCST: Wisconsin Card Sorting Test
TEN: Total error number
CEN: Continuous error number
NCEN: Non- continuous error number
VFT: Verbal Fluency Test
VFTFM: First few minutes of the VFT
PYY: Peptide YY
GLP-1: Glucagon-like peptide-1
IL: Interleukin
TNF: Tumor necrosis factor
GABA: γ-aminobutyric acid
DA: Dopamine
Ach: Acetylcholine
ACTH: Adrenocorticotropic hormone
